# Minimally Invasive Treatment of Left Renal Staghorn Calculus After Orthotopic Neobladder Surgery: A Case Report and Literature Review

**DOI:** 10.1002/ccr3.71029

**Published:** 2025-10-17

**Authors:** Xiaozhi Shi, Wei Li, Fei Gao, Yongan Wen

**Affiliations:** ^1^ Department of Urology The Affiliated Hospital of Xizang Minzu University Xianyang China; ^2^ Department of Internal Medicine The Affiliated Hospital of Xizang Minzu University Xianyang China; ^3^ Department of Hematology The Sixth Affiliated Hospital of Jinan University Dongguan China

**Keywords:** case report, orthotopic neobladder, retroperitoneal laparoscopic pyelolithotomy, staghorn calculi

## Abstract

A 60‐year‐old male with left renal staghorn calculi post orthotopic neobladder surgery was treated via retroperitoneal laparoscopic pyelolithotomy plus flexible ureteroscopic holmium laser lithotripsy, achieving full stone clearance.

## 1 Introduction

Bladder cancer is one of the most common malignant tumors of the urinary system worldwide, second only to prostate cancer, with an annual incidence rate of 20 per 100,000 in men and 5 per 100,000 in women [1]. The treatment of bladder cancer primarily depends on the tumor stage and pathological type, and approximately 25%–30% of patients are diagnosed with locally invasive bladder cancer, which typically requires radical cystectomy followed by urinary diversion to restore urinary function [2]. Renal calculi are a common late complication of radical cystectomy and urinary diversion [3], particularly with a relatively high incidence after orthotopic neobladder surgery [4]. The development of renal calculi after orthotopic neobladder surgery is closely related to changes in urinary tract anatomy. These changes lead to altered urodynamics and bladder dysfunction, which cause poor urine flow [5]. Here, we report a case of left renal calculi occurring 6 years after orthotopic neobladder surgery. We report the diagnosis and treatment process of this case to share our experience. Additionally, we conduct a literature review to provide clinical reference for managing similar complex cases.

## 2 Case Presentation

### 2.1 Patient Background

A 60‐year‐old male was admitted to the hospital on July 4, 2024, with the chief complaint of “painless gross hematuria for 1 week after 6 years of orthotopic neobladder surgery.” Six years prior, the patient underwent laparoscopic radical cystectomy and orthotopic neobladder surgery for bladder malignancy. One week before admission, the patient experienced one episode of gross hematuria during urination without an obvious trigger, which resolved after increased water intake. Past Medical History: The patient had no significant comorbidities and was not taking any nephrotoxic or stone‐related medications.

### 2.2 Laboratory and Imaging Examinations

#### 2.2.1 Laboratory Examinations

Urine routine test showed occult blood (+) and leukocyte (+). Urine culture was negative. Renal function tests revealed a serum creatinine level of 78.8 μmol/L and an estimated glomerular filtration rate (eGFR) of 92.43 mL/(min·1.73 m^2^).

#### 2.2.2 Imaging Examination

**FIGURE 1:**
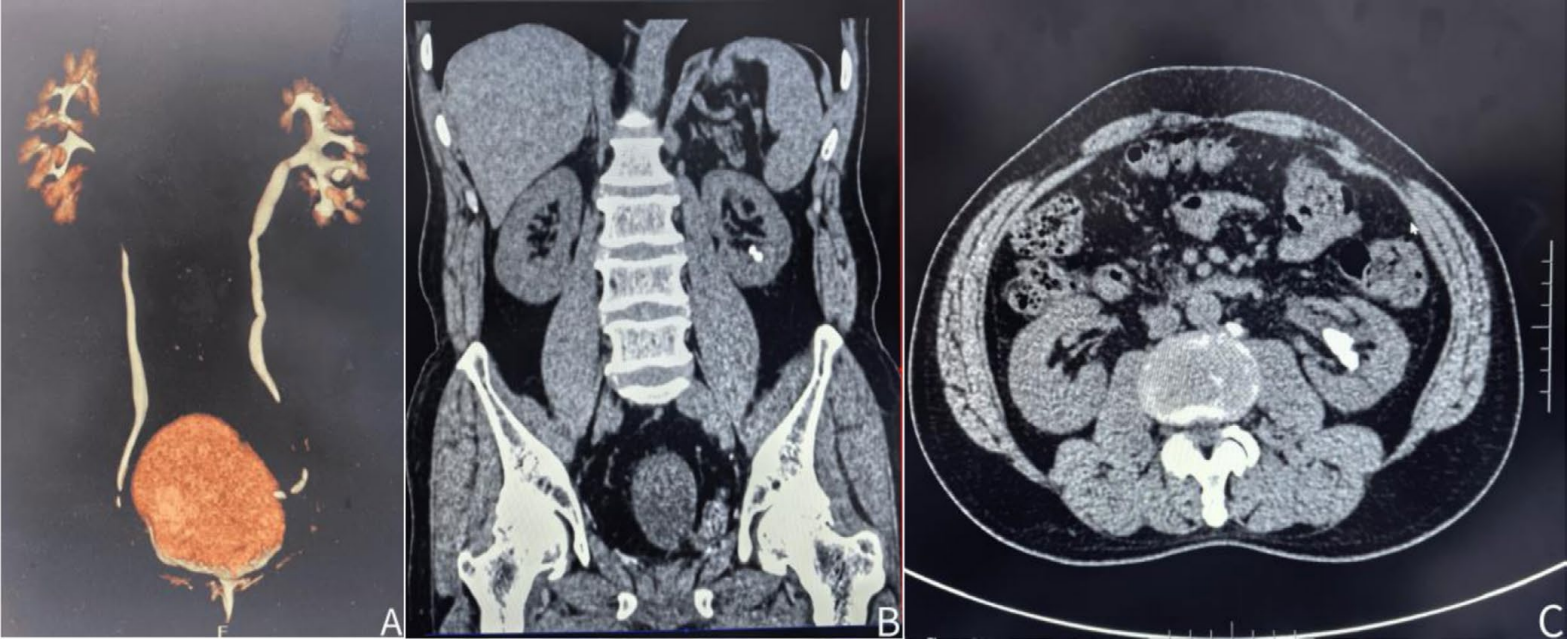
Preoperative computed tomography urography, (A) Three‐dimensional reconstruction demonstrates the ureteral orifice positioned at the bladder apex, (B) Sagittal view reveals the renal calculus in the lower calyceal, (C) Coronal view of CTU shows the calculus located in the renal pelvis.

Computed Tomography Urography (CTU) demonstrated post‐orthotopic neobladder changes, left renal calculi, irregular bladder morphology, and absence of the prostate and bilateral seminal vesicles (Figure 1).

### 2.3 Preoperative Evaluation

#### 2.3.1 Stone Characteristics

Size: 2.4 × 1.1 cm.

Area: 2.07 mm^2^.

Location: left renal pelvis and lower calyceal.

CT value: 1465 HU.

#### 2.3.2 Perioperative Infection Risk Assessment

Low‐risk group (negative urine culture, afebrile, no hydronephrosis).

Renal hydronephrosis status: No hydronephrosis.

Renal pelvis anatomy: Intrarenal pelvis.

Ureteral orifice location: Reimplanted ureter, located at the apex of the bladder.

### 2.4 Surgical Procedure

Under general anesthesia, the patient was placed in a lithotomy position. A cystoscope was inserted through the urethra, but the left ureteral orifice could not be identified, and guidewire insertion failed. Consequently, the patient's position was then changed, and a retroperitoneal laparoscopic pyelolithotomy was performed, together with flexible ureteroscopic laser lithotripsy and placement of a double‐J stent.

**FIGURE 2:**
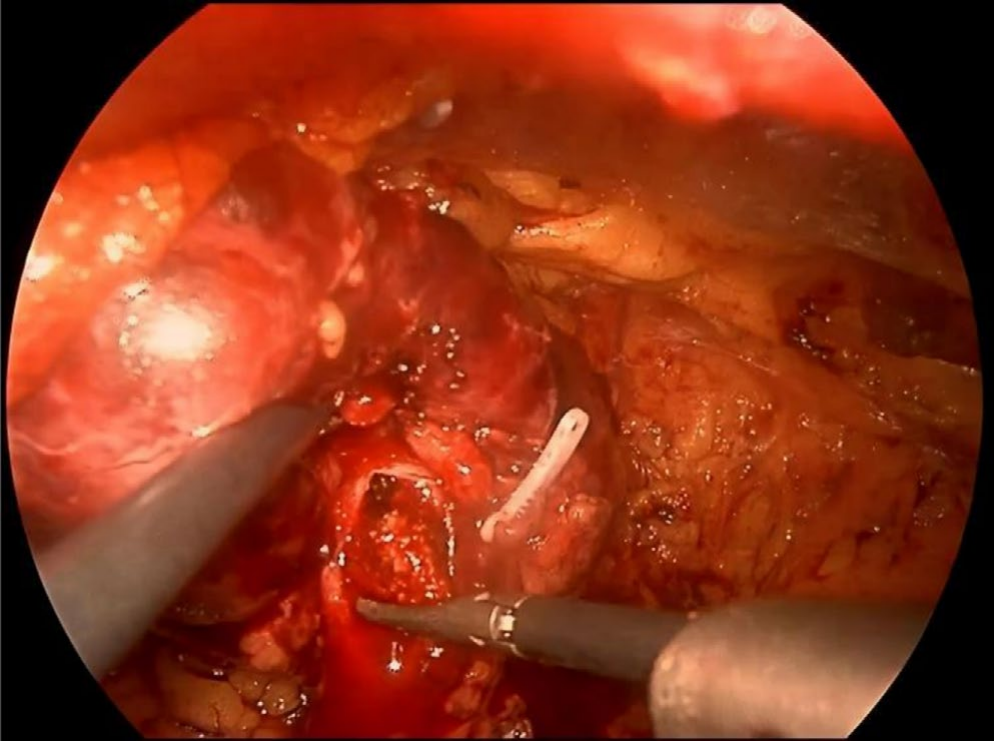
Retroperitoneal laparoscopic pyelolithotomy—extraction of renal pelvis stones using a grasper.

**FIGURE 3:**
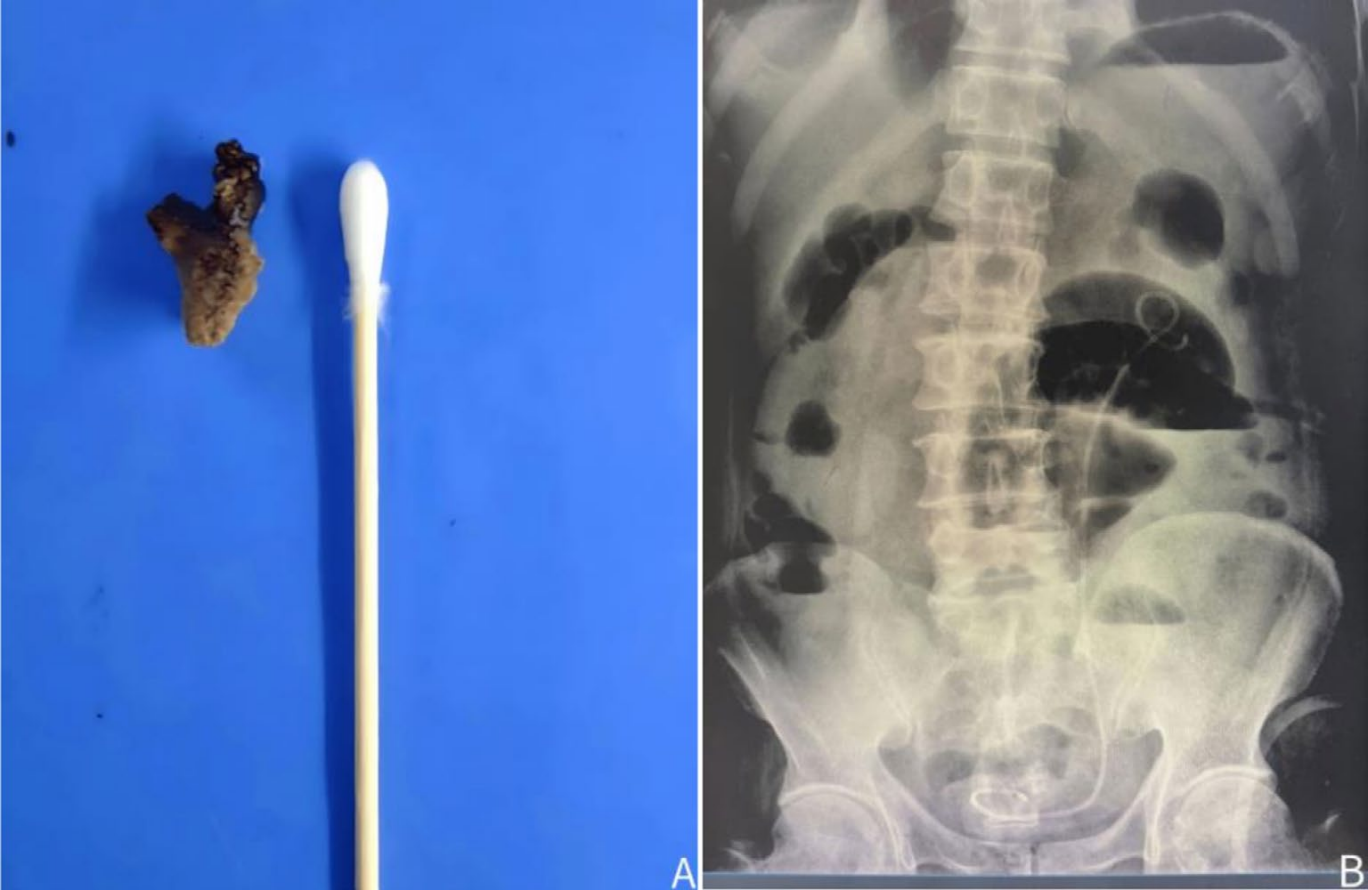
Postoperative follow‐up, (A) Retrieved renal pelvic calculi, (B) KUB showing double‐J stent in the left ureter.

Using an ultrasonic knife, the lower pole of the left kidney was fully mobilized, and dissection was carried upward along the ureter to the renal pelvis. The posterior lip of the inferior calyces was incised with an ultrasonic knife. Separating pliers were used to explore and extract the calculus from the renal pelvis (Figure 2). Residual calculi in the lower calyces were fragmented with holmium laser lithotripsy under flexible ureteroscopic guidance. All targeted calculi were successfully removed (Figure 3A). A double‐J stent was inserted, and the incision was sutured with 4–0 absorbable barbed sutures. Immediate postoperative KUB plain film confirmed that the double‐J stent was in place (Figure 3B). The operation lasted 250 min, with an estimated blood loss of 50 mL. The patient was safely transferred back to the ward after the procedure.

## 3 Discussion

Orthotopic neobladder surgery is a common urinary diversion and reconstruction procedure following radical cystectomy [6]. Due to the neobladder being a reconstructed urinary reservoir and the positional shift of ureteral orifices, managing renal calculi in such patients presents significant challenges. Through domestic and international literature searches, we failed to find medical report records that completely match the characteristics of this case. In light of this, we systematically analyzed the diagnostic and treatment strategies for this type of disease by reviewing recent relevant literature and integrating our diagnostic and treatment processes for this case, with the aim of providing a reference for clinical practice.

Surgical challenges in this case: (1) The altered anatomy of the orthotopic neobladder posed a critical challenge in identifying the ureteral orifice, which could have resulted in failed ureteroscope insertion. (2) A non‐hydronephrotic renal pelvis leads to poor visibility of the percutaneous nephrolithotomy (PCNL) puncture target, thereby increasing the risk of puncture failure. Even with successful puncture, the risk of double‐J stent placement failure was elevated due to anatomical distortion of the ureter and the potential for stricture at the uretero‐neobladder anastomosis site. (3) The deeply positioned intrarenal pelvis required a deep pyelotomy incision during laparoscopy, exposing the patient to an increased risk of renal vascular injury. (4) Challenges with stone clearance rates.

Experience sharing: (1) In cases of renal or ureteral calculi complicating orthotopic neobladder surgery where ureteral orifices cannot be identified, laparoscopic lithotomy offers a viable solution. This approach avoids the challenges associated with failed double‐J stent placement postoperatively. (2) When encountering an intrarenal pelvis, the avascular plane of the extra‐pelvic space allows for safe dissection. The free anastomosis of intrarenal veins ensures that transection of these vessels does not compromise renal parenchymal blood flow, permitting a “cut‐and‐suture” technique. Additionally, longitudinal incisions along the calyceal neck, parallel to the muscle fibers, help preserve postoperative peristaltic function [7]. (3) The posterolateral calyceal neck incision is designed along the avascular plane defined by renal arterial blood supply, minimizing the risk of segmental artery injury [8]. (4) For lower calyceal calculus, complete renal mobilization followed by caudal retraction of the lower pole optimizes flexible ureteroscopic visualization.

Notably, we chose retroperitoneal laparoscopic pyelolithotomy combined with flexible ureteroscopic holmium laser lithotripsy based on a critical comparison of alternative surgical options. This choice specifically addressed treatment for post‐orthotopic neobladder renal calculi. PCNL, a first‐line approach for complex calculi in general populations, was deemed infeasible here. The patient's non‐hydronephrotic intrarenal pelvis reduced puncture target visibility, elevating the risk of renal injury, and distorted ureteral anatomy complicated double‐J stent placement. Alternatively, robotic‐assisted laparoscopic pyelolithotomy, while offering enhanced dexterity, lacked universal availability, and both cost and limited accessibility further restricted its utility. In contrast, our combined minimally invasive strategy addressed anatomical barriers without PCNL's puncture risks or the resource constraints associated with robotic techniques. This approach achieved complete stone clearance with minimal blood loss and no complications, underscoring its suitability for such complex cases.

In this case, retroperitoneal laparoscopic pyelolithotomy combined with flexible ureteroscopic laser lithotripsy was performed, and complete stone clearance was achieved. While laparoscopic pyelolithotomy effectively addressed the renal pelvis and middle calyceal calculi, complete clearance of lower calyceal calculi was challenging due to the limited accessibility of the rigid laparoscopic approach. Flexible ureteroscopy, although useful for fragmenting lower calyceal calculi, faced limitations in stone retrieval due to the acute infundibular‐pelvic angle and restricted maneuverability, potentially leading to residual fragments.

Clinically, complex renal calculi present diverse challenges, requiring personalized treatment strategies based on individual anatomical variations, stone characteristics, and patient comorbidities. This case highlights the value of combined minimally invasive techniques while acknowledging their limitations in cases of extreme anatomical variants.

## Author Contributions

**Xiaozhi Shi:** supervision, validation, visualization, writing – original draft, writing – review and editing. **Wei Li:** writing – original draft, writing – review and editing. **Fei Gao:** writing – original draft, writing – review and editing. **Yongan Wen:** conceptualization, data curation, writing – review and editing.

## Ethics Statement

All studies contributing data to these analyses had the relevant institutional review board approval from each country, the Institutional Review Board of Xizang Minzu University in accordance with the Declaration of Helsinki.

## Consent

Written informed consent was obtained from the patient to publish this report in compliance with the journal's patient consent policy.

## Conflicts of Interest

The authors declare no conflicts of interest.

## Data Availability

The data presented in this study is available within the article.

## References

[1] Y. Fan , X. Li , H. Sun , Z. Gao , Z. Zhu , and K. Yuan , “Role of WTAP in Cancer: From Mechanisms to the Therapeutic Potential,” Biomolecules 12, no. 9 (2022): 1224. 36139062 10.3390/biom12091224 PMC9496264

[2] J. Yu , W. Mao , S. Sun , et al., “Characterization of an Autophagy‐Immune Related Genes Score Signature and Prognostic Model and Its Correlation With Immune Response for Bladder Cancer,” Cancer Management and Research 14 (2022): 67–88. 35023971 10.2147/CMAR.S346240 PMC8743383

[3] F. Presicce , C. Leonardo , G. Tuderti , et al., “Late Complications of Robot‐Assisted Radical Cystectomy With Totally Intracorporeal Urinary Diversion,” World Journal of Urology 39, no. 6 (2021): 1903–1909. 32747981 10.1007/s00345-020-03378-7 PMC8217047

[4] B. Zhang , H. Xie , and C. Liu , “Risk Factors of Calculi in Upper Urinary Tract After Radical Cystectomy With Urinary Diversion,” Actas Urológicas Española 43, no. 10 (2019): 568–572. 10.1016/j.acuro.2019.04.002 31358300

[5] J. Gu , Z. He , H. Li , et al., “A Giant Neobladder Stone With Insignificant Symptoms: A Case Report and Literature Review,” Frontiers in Surgery 10 (2023): 1105146. 36874453 10.3389/fsurg.2023.1105146 PMC9977997

[6] Q. Luo , L. Xu , C. Lin , H. Chao , T. Zeng , and Z. Zhu , “The Clinical Study of Urinary Flow Parameters After Drag‐And‐Bond Anastomosis for Ileal Orthotopic Neobladder Reconstruction,” International Urology and Nephrology 56, no. 8 (2024): 2615–2621. 38502467 10.1007/s11255-024-04015-7

[7] L. Hu , N. Zhang , X. Zhang , H. Liang , Y. Fan , and J. Chen , “Laparoscopic Pyelotomy Combined With Ultrasonic Lithotripsy via a Nephroscope for the Treatment of Complex Renal Stones,” Urolithiasis 52, no. 1 (2024): 22. 38189842 10.1007/s00240-023-01522-7

[8] R. K. Kaushik , S. Khare , S. Jain , A. Tripathi , H. Kausar , and S. Raizaday , “Study of Renal Posterior Segmental Artery by Corrosion Cast Method,” Journal of Evidence‐Based Medicine and Healthcare 7 (2020): 2600–2603.

